# Estrogen receptor β is associated with expression of cancer associated genes and survival in ovarian cancer

**DOI:** 10.1186/s12885-018-4898-0

**Published:** 2018-10-16

**Authors:** Susanne Schüler-Toprak, Florian Weber, Maciej Skrzypczak, Olaf Ortmann, Oliver Treeck

**Affiliations:** 1 Department of Obstetrics and Gynecology, University Medical Center Regensburg, Landshuter Str. 65, 93053 Regensburg, Germany; 2 Department of Pathology, University Medical Center Regensburg, Franz-Josef Strauß Allee 11, 93053 Regensburg, Germany; 3 Second Department of Gynecology, Medical University of Lublin, Jaczewskiego 8, 20-090 Lublin, PL Poland

**Keywords:** Estrogen receptor β, Ovarian cancer, Overall survival, Progression-free survival

## Abstract

**Background:**

In ovarian cancer, the role of estrogen receptors (ERs), particularly of ERβ, being suggested as tumor suppressor in breast and prostate cancer, remains unclear. We examined the expression of nuclear and cytoplasmic ERβ in ovarian cancer and correlated it with expression of ovarian cancer markers CA125, CEA and CA72–4, steroid hormone receptors ERα and PR, cancer-associated genes EGFR, p53, HER2 and proliferation marker Ki-67. Additionally we examined to what extent expression of ERβ and the other proteins affects survival of ovarian cancer patients.

**Methods:**

We established a tissue microarray from 171 ovarian cancer patients and performed immunohistochemical analyses of the mentioned proteins.

**Results:**

Nuclear ERβ was detected in 47.31% of the ovarian cancer tissues and cytoplasmic expression of this receptor was observed in 23.08%. Nuclear expression of ERβ was significantly decreased in the G3 subgroup compared to better differentiated cancers (*p* <  0.01) and correlated with ovarian cancer markers CEA (95% CI 0.1598–0.4465; *p* <  0.0001) and CA72–4 (95% CI 0.05953–0.3616; *p* <  0.01). Cytoplasmic ERβ expression correlated with EGFR levels (95% CI 0.1059–0.4049; *p* <  0.001). ERα expression was associated with expression of CA125 and PR. Overall survival of patients with tumors expressing cytoplasmic ERβ was significant longer compared to those with ERβ-negative ovarian cancer (chi-square statistic of the log-rank, *p* < 0.05). Progression-free survival was dependent on expression of PR (chi-square statistic of the log-rank, *p* < 0.05) and Ki-67 (*p* = 0.05).

**Conclusions:**

Our data suggest an important, but distinct role of nuclear and cytoplasmic ERβ expression in ovarian cancer and encourage further studies on its role in this cancer entity.

**Electronic supplementary material:**

The online version of this article (10.1186/s12885-018-4898-0) contains supplementary material, which is available to authorized users.

## Background

Ovarian cancer is the leading cause of death from a gynecological malignancy in the developed world [1]. Ovarian cancers are influenced by steroid hormones. Antiestrogenic treatment inhibits growth of ovarian cancer in vitro and in vivo [2–4]. Progesterone receptor (PR) and ER expression are reported to be associated with improved ovarian cancer survival, independent of clinical prognostic factors, but these associations have not been consistently repeated [5, 6]. In the clinical setting antiestrogens are commonly used in patients with relapsed ovarian cancers after multiple lines of cytostatic therapies that have exhausted further treatment. Available data remains inconclusive, mostly due to heterogeneity of ovarian cancers and inadequate study settings as low number of included patients, missing subgroup analyses and absent evaluation of ER expression. However, use of tamoxifen and aromatase inhibitors have effects in a certain subgroup of patients [7, 8].

Previous studies clearly suggest a tumor suppressive role of ERβ in ovarian cancer as it has been shown for breast or prostate cancer [9–11]. Our group demonstrated that ERβ reduces proliferation and migration, but activates apoptosis of ovarian cancer cells [12] and that specific ERβ-agonists significantly inhibit growth of different ovarian cancer cell lines [13]. Furthermore, our results from a phenotype-genotype association study suggested that the single nucleotide polymorphism rs3020449 in the promoter region of *ESR2* gene might affect progression of ovarian cancer [14].

ERβ is the predominant ER in normal ovarian tissue. In ovarian cancer the expression of ERβ is significantly lower and the ERα/ERβ ratio is significantly higher than in normal ovarian tissue [15–17]. High expression of ERβ in ovarian cancers is associated with a better progression-free and overall survival [15, 17].

Ovarian cancer marker cancer antigen 125 (CA125) is overexpressed in the majority of ovarian cancers and it has been shown to be involved in the metastatic process [18]. CA125 regulates cell adhesion by interacting with mesothelin, galectin-1, E-cadherin and β-catenin [18, 19]. Moreover, CA125 can promote proliferation and migration [20]. Also for the broad spectrum tumor marker carcinoembryonic antigen (CEA) a regulative role of cell adhesion and thus an influence on metastasis of cancer cells has been suggested [21]. Another antigen selectively expressed in ovarian cancer is CA72–4. As it is highly detectable in all ovarian cancer subtypes it is another effective tumor marker in ovarian cancer [22]. However, its function in carcinogenesis of ovarian cancer is still unknown.

Recently, it has been reported that a shuffle of ERβ between nucleus and cytoplasm plays important roles in regulation of gene transcription, RNA maturation and post-transcriptional control [23]. To further approach the significance of subcellular ERβ localization in ovarian cancer, we examined co-expression of nuclear and cytoplasmic ERβ with various cancer-associated genes and steroid hormone receptors and tested to what extent receptor localization would affect survival of ovarian cancer patients.

## Methods

### Tissue samples

We included ovarian cancer samples collected in the Department of Gynecology and Obstetrics of the University of Regensburg. Caucasian women with sporadic ovarian cancer and available information on grading, stage, and histological subtype from 1995 to 2013 were included. Data from the Tumor Centre Regensburg (Bavaria, Germany), a high-quality population-based regional cancer registry of the districts of Upper Palatinate and Lower Bavaria, were analysed. Mortality data were obtained from regional registration offices. The institutional review board “Ethikkommission der Universität Regensburg” approved the retrospective study.

### Tissue microarray and immunohistochemistry

The tissue microarray (TMA) was created using standard procedures that have been previously described [24]. From all patients included in this study, an experienced pathologist evaluated H&E sections of tumor tissue and representative areas were marked. From each tumor, one single core was included in the final TMA. From these areas, core biopsies on the corresponding paraffin blocks were removed and transferred into the grid of a recipient block according to a predesigned array of about 60 specimens in each of five TMA paraffin blocks.

For immunohistochemistry, 4 μm sections of the TMA blocks were incubated with the indicated antibodies **(**Additional file 1) according to routine protocols in the given dilutions, followed by incubation with an HRP-conjugated secondary antibody and another incubation with 3,3′-diaminobenzidine (DAB) as substrate, which resulted in a brown-colored precipitate at the antigen site. An experienced clinical pathologist evaluated immunohistochemical staining according to localization and specificity. For steroid hormone receptors ERα, nuclear ERβ and PR, the immunoreactivity score (IRS) according to Remmele et al. was used [25], wherein the percentage of stained nuclei in a 5-tiered scale (0% = 0, 1–9% = 1, 10–50% = 2, 51–80% = 3 and 81–100% = 4) is multiplied with the staining intensity on a 4-tiered scale (no staining = 0, weak staining = 1, moderate staining = 2, strong staining = 3), resulting in an IRS ranging between 0 (completely negative) and 12 (strongly positive).

Expression of proliferation marker Ki-67 using antibody clone MIB-1 was assessed in the percentage of tumor cells with positive nuclear staining. Her2/neu expression was scored according to the DAKO score routinely used for breast cancer cases. EGFR was scored according to Spaulding et al. [26] on a 4-tiered scale from 0 to 3. EGFR expression was considered present when membranous staining was stronger than unspecific or cytoplasmic background staining, irrespective of complete or incomplete circumferential staining. Score 1 was defined as incomplete and weak membranous staining in > 1% of tumor cells, score 2 as moderate staining and score 3 as strong membranous staining in > 1% of tumor cells.

For p53 and polyclonal CEA, the “quickscore” was used, where results are scored by multiplying the percentage of positive cells (P) by the intensity (I) according to the formula: Q = P x I; maximum = 300 [27]. CA-125 and cytoplasmic ERβ were described as positive or negative, irrespective of staining intensity. CA72–4 expression was assessed on a 4-tiered scale, wherein no expression equaled score 0, weak cytoplasmic and/or membranous staining in > 1% of tumor cells equaled score 1, moderate staining score 2 and strong staining corresponded to score 3 (if > 50% of tumor cells, otherwise score 2).

### Statistical analysis

Statistical analysis was performed using GraphPad Prism 5® (GraphPad Software, Inc., La Jolla, CA, USA). The non-parametric Kruskal-Wallis rank-sum test was used for testing differences in receptor expression among three or more groups. For pairwise comparison the nonparametric Mann-Whitney-U rank-sum test was used. Correlation analysis was performed using the Spearman correlation coefficient. The chi-square statistic of the log-rank was used to investigate differences between survival curves. *P*-values below 0.05 were considered statistically significant.

## Results

### Characteristics of included patients and their tumors

In this study, we used tissue from 171 Caucasian women with sporadic ovarian cancer and a median age at diagnosis of 63.5 years (range 29–91). The histopathological characteristics of the patients are shown in Table 1. 63.7% of the tumors were diagnosed in FIGO (International Federation of Gynaecologists and Obstetricians) stages III and IV (38.6 and 25.15, respectively). Most of the tumors were serous (78.36%) and 64.3% of the tumors were grade 3. Median follow-up time was 1180 days. 80 relapses and 62 deaths were documented. Median relapse free survival was 1044 days and median overall survival was 1673 days.

**Table 1:** Staging and histopathological characteristics of ovarian cancer cases

Characteristics	Number of patients	(%)
Ovarian cancer patients	171	
FIGO stage		
FIGO I	23	(13.45)
FIGO II	9	(5.26)
FIGO III	66	(38.60)
FIGO IV	43	(25.15)
unknown	30	(17.54)
Histological subtype		
serous	134	(78.36)
mucinous	6	(3.51)
endometrioid	10	(5.85)
clear cell	5	(2.92)
undifferentiated	16	(9.36)
Histological grade		
G2	47	(27.49)
G3	110	(64.33)
unknown	14	(8.19)

### Expression of steroid hormone receptors in ovarian cancer tissue

**Fig. 1:**
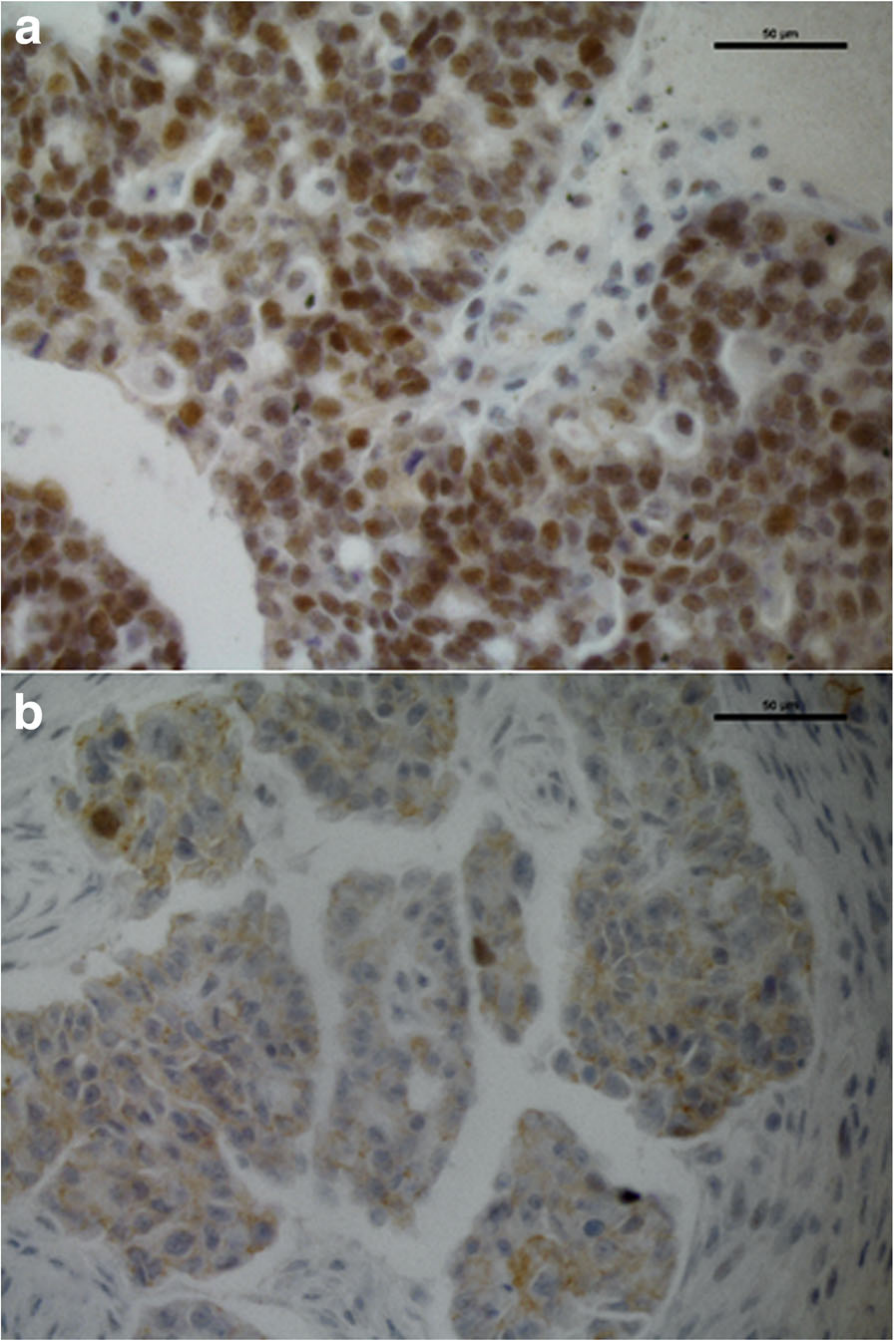
Immunohistochemical staining patterns of ERβ: **a** Moderate to strong nuclear expression of ERβ. **b** Weak cytoplasmic expression of ERβ

**Fig. 2:**
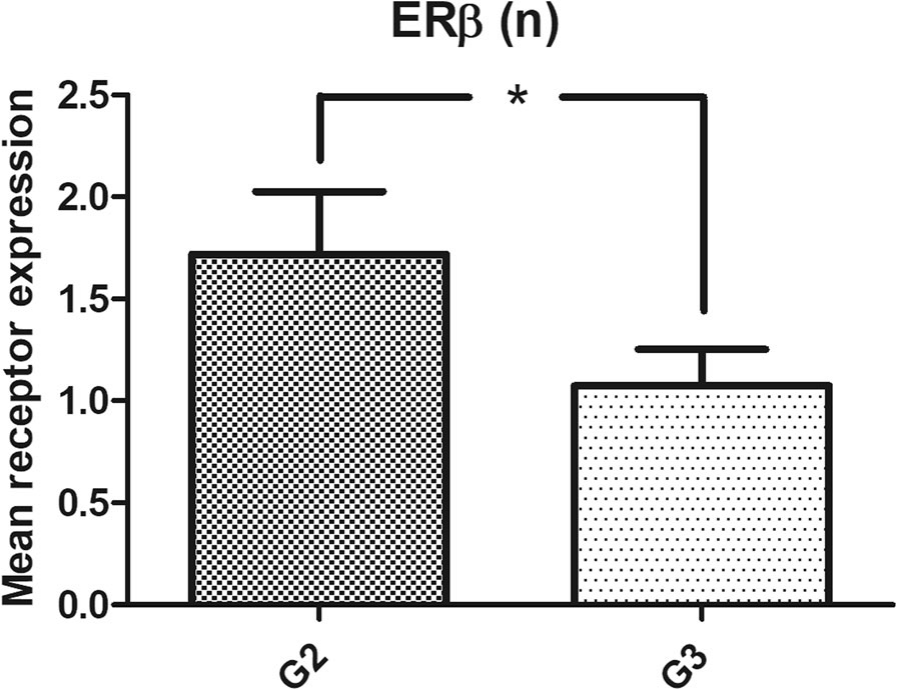
Expression of nuclear ERβ in G2 and G3 graded ovarian cancer. Shown are the mean values of the immunoreactivity scores (*p* < 0.05). n (G2) = 64, n (G3) = 109

Nuclear ERβ was expressed in 47.31%, and cytoplasmic expression of this receptor was detected in 23.08% of ovarian cancer specimens (Fig. 1**,** Table 2). Nuclear ERβ expression was found to be lower in the subgroup of G3 tumors than in better differentiated cancer specimens (*p* < 0.05) (Table 3, Fig. 2). ERα was expressed in 70.35% of all ovarian cancer samples, whereas PR was detected in 33.33% of the samples. PR expression was higher in FIGO I + II tumors than in the FIGO III + IV subgroup (*p* < 0.05) (Table 3**)**. A significantly higher expression of PR was also found in tumors without invasion of lymphatic vessels compared to those with invasion. As serous ovarian cancer was the most common histological subtype, we further analysed this group and found similar rates of positive receptor expression. The subgroup of G3-tumors and those of stage III and IV also did not show significant differences. Further subgroup-analyses as well as analyses of other variables as residual disease after initial surgery, age at diagnosis or invasion of venous vessels did not reveal significant results because of the low number of included cases.

**Table 2:** Steroid hormone receptor expression in ovarian cancer: rate of expression of the indicated receptors

		ERα	ERβ (nuclear)	ERβ (cytoplasmic)	PR
All	All	121/172 (70.35)	79/167 (45.04)	39/169 (23.08)	55/165 (33.33)
	G2	33/47 (70.21)	29/46 (63.04)	12/46 (26.09)	15/47 (31.91)
	G3	78/111 (70.27)	45/109 (41.28)	19/109 (17.43)	36/106 (33.96)
	FIGO I + II	21/32 (70.54)	12/30 (40.00)	7/31 (22.58)	16/31 (51.61)
	FIGO III + IV	79/112 (65.63)	45/100 (45.00)	7/31 (22.58)	31/108 (28.70)
Serous	Serous	99/133 (74.44)	59/131 (49.62)	27/131 (20.61)	46/128 (35.94)
	G2	23/31 (74.19)	18/31 (58.06)	9/31 (29.03)	11/31 (35.48)
	G3	71/97 (73.20)	38/95 (40.00)	17/95 (17.89)	33/92 (35.87)
	FIGO I + II	15/20 (75.00)	5/19 (26.32)	6/20 (30.00)	12/19 (63.16)
	FIGO III + IV	70/96 (72.92)	45/95 (47.37)	19/92 (20.65)	28/92 (30.43)

Shown are the numbers of positive samples in relation to the total numbers of ovarian cancer cases of the subgroups analysed and the corresponding percentage (in brackets)

**Table 3:** Steroid hormone receptor expression in ovarian cancer: mean receptor expression levels in all ovarian cancer specimens

	ERα		ERβ (nuclear)		ERβ (cytoplasmic)		PR	
	Mean	*p*-value	Mean	*p*-value	Mean	*p*-value	Mean	*p*-value
G2	4.30	0.9464	1.72	**0.0466**	0.26	0.4424	2.19	0.9202
G3	4.06		1.07		0.17		1.19	
FIGO I + II	3.97	0.9172	1.27	0.6752	0.23	0.9548	3.48	**0.0202**
FIGO III + IV	4.15		1.28		0.21		1.06	
G2	4.26	0.9835	1.58	0.2049	0.29	0.4139	1.90	0.8961
G3	4.06		1.12		0.18		1.18	
FIGO I + II	3.80	0.9338	0.79	0.2563	0.30	0.6205	3.37	**0.0190**
FIGO III + IV	4.10		1.31		0.21		1.05	

A non-parametric Kruskal-Wallis rank-sum test was used for testing differences in receptor expression of estrogen receptor (*ER*) α, nuclear ERβ, cytoplasmic ERβ and progesterone receptor (*PR*) among the groups. *p*-values < 0.05 were considered statistically significant (indicated by using bold font)

### Nuclear ERβ expression levels positively correlate with expression of CEA and CA72–4

Subsequently, we investigated correlations between the expression levels of ERβ and ERα, PR, CA125, CEA, CA72–4, EGFR, HER2, Ki-67 and p53.

We found a highly significant positive correlation between nuclear ERβ and ovarian cancer marker CEA (*p* < 0.0001; 95% confidence interval (CI) 0.1598–0.4465) (Table 4). This significant correlation was also found in the serous subtype (*p* < 0.0001; 95% CI 0.1770–0.4930). Moreover, we observed a highly significant correlation between nuclear ERβ and CA72–4 (*p* < 0.01; 95% CI 0.05953–0.3616) in all ovarian cancer specimens as well as in the serous subtype (*p* < 0.01; 95% CI 0.08939 to 0.4225).

**Table 4:** Correlation of nuclear ERβ expression in ovarian cancer with expression of ovarian cancer markers, cancer-associated genes and steroid hormone receptors

		CA125	CEA	CA72–4	EGFR	HER2	Ki-67	P53	ERα	PR
All	Spearman r	−0.041	**0.31**	**0.216**	0.054	0.045	−0.084	0.101	0.086	0.006
	95% CI	−0.198 - 0.117	**0.16** **- 0.447**	**0.06** **- 0.362**	−0.107 - 0.213	− 0.114 - 0.201	− 0.246 - 0.083	− 0.058 - 0.255	− 0.073 - 0.240	− 0.154 - 0.166
	*P* value	n.s.	**< 0.0001**	**0.0057**	n.s.	n.s.	n.s.	n.s.	n.s.	n.s.
Serous	Spearman r	0.051	**0.345**	**0.264**	0.089	0.098	−0.061	0.067	0.095	0.011
	95% CI	−0.129 - 0.227	**0.177** **- 0.493**	**0.089** **- 0.423**	−0.0940 - 0.266	−0.083 - 0.274	− 0.245 - 0.128	−0.113 - 0.242	− 0.084 - 0.269	−0.170 - 0.193
	*P* value	n.s.	**< 0.0001**	**0.0026**	n.s.	n.s.	n.s.	n.s.	n.s.	n.s.

Correlation of nuclear ERβ expression with expression of ovarian cancer markers CA125, CEA, CA72–4, and with EGFR, HER2, Ki-67, P53, ERα and PR were calculated for all ovarian cancers (“all”) and serous ovarian cancers (“serous”) using the Spearman correlation coefficient. *P*-values below 0.05 were considered statistically significant (significant results were indicated by using bold font)

*CI* confidence interval

### Cytoplasmic ERβ expression levels positively correlate with EGFR expression

Additionally, we observed a significant correlation between cytoplasmic ERβ expression levels with expression of EGFR (*p* < 0.001; 95% CI 0.1059–0.4049). In serous ovarian cancers this effect reappeared (*p* < 0.01; 95% CI 0.06–0.4016) and we observed a significant correlation with PR expression (*p* < 0.05; 95% CI 0.02307–0.3688) (Table 5).

**Table 5:** Correlation of cytoplasmic ERβ with expression of cancer-associated genes and steroid hormone receptors

		CA125	CEA	CA72–4	EGFR	HER2	Ki-67	P53	ERα	ERβ (n)	PR
All	Spearman r	0.073	0.036	0.026	**0.262**	0.089	−0.012	0.037	−0.049	−0.085	0.131
	95% CI	−0.086 − 0.227	−0.122 − 0.193	−0.132 - 0.183	**0.106** **- 0.405**	−0.069 - 0.243	− 0.175 - 0.152	−0.121 - 0.194	− 0.205 - 0.109	−0.238 - 0.073	− 0.029 - 0.284
	*p* value	n.s.	n.s.	n.s.	**0.0009**	n.s.	n.s.	n.s.	n.s.	n.s.	n.s.
Serous	Spearman r	−0.007	0.041	−0.026	**0.238**	0.020	0.174	0.083	−0.109	−0.078	**0.202**
	95% CI	−0.183 - 0.17	−0.138 - 0.216	− 0.202 − 0.152	**0.06** **− 0.402**	−0.158 - 0.197	− 0.010 - 0.346	−0.095 - 0.257	− 0.281 - 0.07	−0.252 - 0.099	**0.023–0.3699**
	*p* value	n.s.	n.s.	n.s.	**0.0075**	n.s.	n.s.	n.s.	n.s.	n.s.	**0.0231**

Correlations of cytoplasmic ERβ expression with expression of ovarian cancer markers CA125, CEA, CA72–4 and with EGFR, HER2, Ki-67, P53, ERα, nuclear ERβ (n) and PR were calculated for all ovarian cancers (“all”) and serous ovarian cancers (“serous”) using the Spearman correlation coefficient. *P*-values below 0.05 were considered statistically significant (significant results were indicated by using bold font)

*CI* confidence interval

### ERα expression levels correlate with expression of CA125 and PR

Examining the correlation of ERα with different markers, we found a significant correlation between this receptor and CA125 (*p* < 0.001; 95% CI 0.1188–0.4068), which was also observed in the serous subtype (*p* < 0.05; 95% CI 0.03457–0.3702). The correlation between ERα and CA125 was also significant in the subgroups of G2 and G3 ovarian cancers (*p* < 0.01; 95% CI 0.1431–0.6367 and *p* < 0.01; 95% CI 0.0567–0.4210, respectively) (Additional file 2).

The expression of ERα significantly correlated with expression of PR (*p* < 0.0001; 95% CI 0.2143–0.4914), which was also observed in the subgroup of serous ovarian cancers (*p* < 0.0009; 95% CI 0.1188–0.4477),

### Overall survival of patients expressing cytoplasmic ERβ is significant longer compared to those of patients with ERβ-negative tumors

**Fig. 3:**
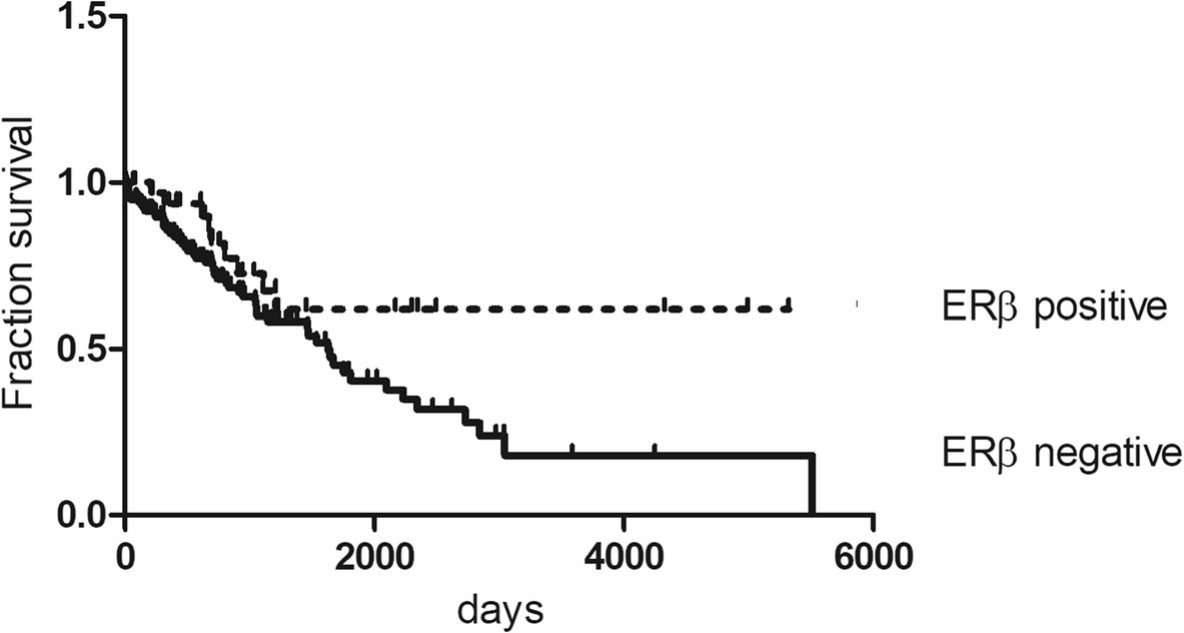
Kaplan Meier survival analysis in ovarian cancer in relation to cytoplasmic ERβ expression. Overall survival of patients with absent expression of cytoplasmic ERβ was compared to survival of patients with cytoplasmic ERβ expression (*p* = 0.0362). The chi-square statistic of the log-rank was used to investigate differences between survival curves. *P*-values below 0.05 were considered statistically significant

Survival analyses revealed a significant longer overall survival of patients with tumors expressing cytoplasmic ERβ compared to those having ERβ-negative tumors (chi-square statistic of the log-rank, *p* < 0.05). Patients with ovarian cancers not expressing cytoplasmic ERβ had a median survival of 1628 days, whereas in the cohort with cytoplasmic ERβ expressing tumors, survival was still more than 50% at the longest time point (hazard ratio (HR) 1.842; 95% CI 1.040–3.264) (Fig. 3).

### Longer progression-free survival of patients with ovarian cancers expressing PR and with low Ki-67 expression

**Fig. 4:**
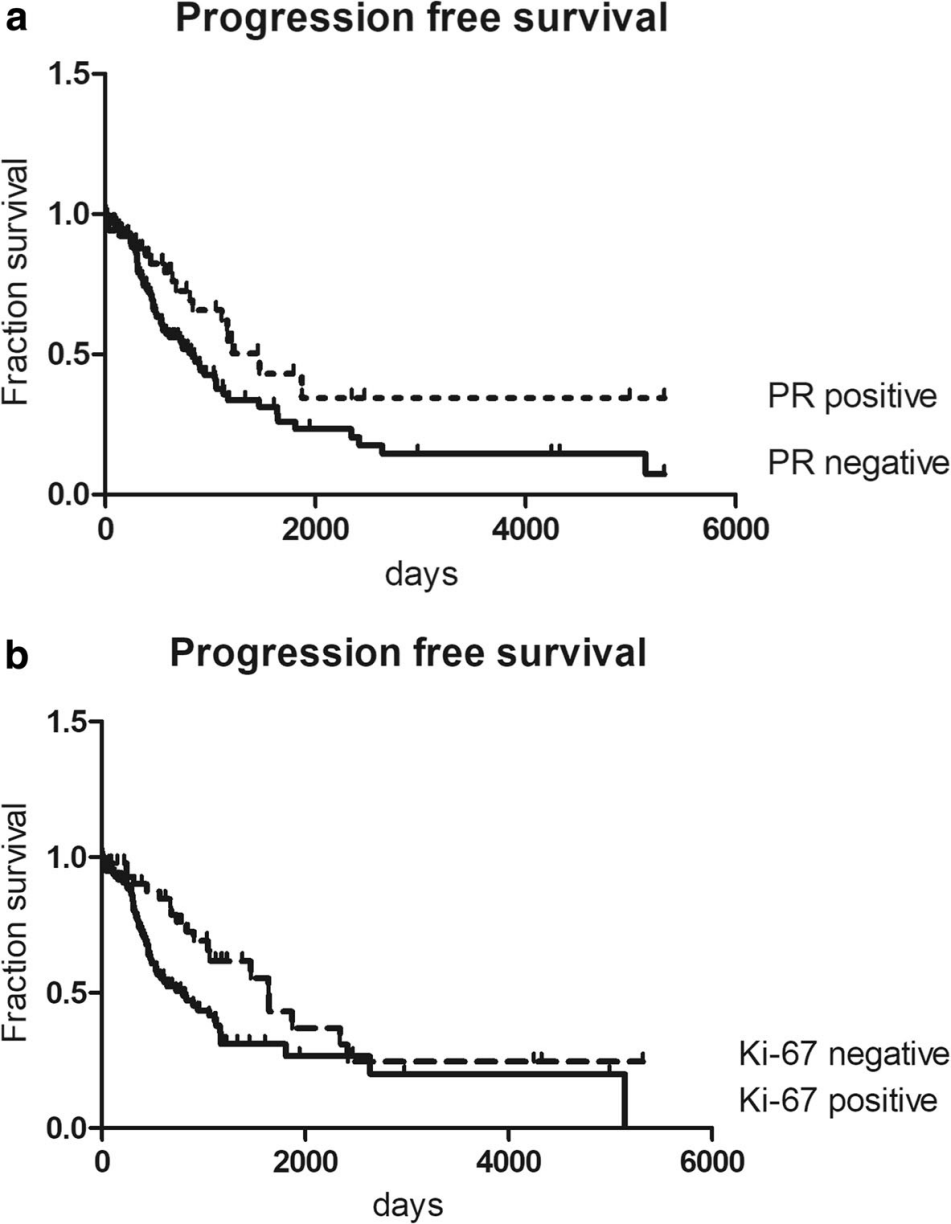
Kaplan Meier survival analysis of progression-free survival (PFS). **a** PFS in all ovarian cancer cases in relation to PR expression. PFS of patients without expression of PR was compared to survival of patients with PR expression (*p* = 0.0261). The chi-square statistic of the log-rank was used to investigate differences between survival curves. *P*-values below 0.05 were considered statistically significant. PFS of those patients whose tumors express PR (IRS ≥ 1) compared to those with PR negative tumors (IRS 0); **b** PFS of ovarian cancer patients whose tumors expressing Ki-67 compared to those with low or absent Ki-67 expression (*p* = 0.0134)

Patients with tumors expressing PR (IRS ≥ 1) had a significant longer progression-free survival compared to those with tumors not expressing PR (IRS 0) (chi-square statistic of the log-rank, *p* < 0.05) (Fig. 4a). This significant difference was also observed in the subgroup of serous ovarian cancers (data not shown). Progression-free survival significantly differed in those patients with tumors expressing Ki-67 by more than 14% (chi-square statistic of the log-rank, *p* < 0.05) (Fig. 4b). This effect also was visible in serous tumors (data not shown).

## Discussion

Several studies showed a wide range of expression levels of steroid hormone receptors in ovarian cancer [28–30]. In our study including 171 ovarian cancer cases, 70.35% of all cases were ERα positive. This is in line with the data of Lee et al. who found this receptor to be expressed in 77.3% of all cases [30]. In other studies, ERα was only expressed in 31.4–36% of ovarian cancer specimens [28, 29]. These differences might be due to different applied ERα antibodies detecting a specific set of splice variants. Investigating ERβ expression reveals even more difficulties as specific antibodies have not been available for a long time. Moreover, it is important to distinguish between nuclear and cytoplasmic ERβ expression as their role in carcinogenesis differs [31, 32]. In our study, nuclear ERβ was expressed in 49.62% and cytoplasmic ERβ in only 20.61% of all cases. Other studies showed rates of positive ERβ expression between 48 and 60% [28, 33]. In the study of De Stefano et al. including 58 serous ovarian cancers nuclear ERβ was expressed in 89.66% and cytoplasmic ERβ was expressed in 77.59% [17].

As expression of ERβ declines during tumorigenesis of breast, colon and prostate cancer, this receptor has been proposed to act as a tumor suppressor [9–11, 34]. ERβ expression is highest in normal ovarian tissue whereas it decreases during dedifferentiation processes [16]. This is in line with our data showing a significantly lower expression of nuclear ERβ in G3 ovarian cancers than in better differentiated tumors. In vitro, we showed reduced proliferation and migration of ovarian cancer cells after overexpression of ERβ as well as increased rates of apoptosis [12]. Moreover, we found a significant growth-inhibition of ovarian cancer cells by treatment with specific ERβ-agonists [13]. Thus, these data support the tumor suppressive role of ERβ in ovarian cancer.

In our study PR expression was significantly higher in early stage ovarian cancers than in cancers with FIGO stages III and IV (*p* = 0.0027). Moreover, we showed a significantly higher expression of PR in tumors without invasion of lymphatic vessels compared to cancers that did not invade lymphatic vessels. In line with data published earlier, progression-free survival of patients with PR-expressing tumors was significantly longer compared to those with tumors not expressing PR [5, 28, 35]. This could be explained by an induction of apoptosis by PR activation in ovarian cancer [5]. Multiple in vitro studies have shown that increased PR expression could promote the progesterone-induced inhibition of DNA synthesis, cell division and proliferation in ovarian cancer cells [35, 36]. Moreover, PR is transactivated by ERα and PR expression may be a biomarker of improved prognosis because it indicates a functionally intact ER pathway and less aggressive tumor behavior [5].

With regard to overall survival and progression-free survival, data from literature remain inconclusive. In our study, patients with tumors expressing cytoplasmic ERβ had a significant benefit in overall survival compared to those with tumors not expressing this form of ERβ. A recent study revealed that ERβ-positive nuclear staining was associated with decreased progression-free survival (hazard ratio (HR) 1.69; 95% CI 0.91–3.15; *p* = 0.096) and decreased overall survival (HR 1.91; 95% CI 0.94–3.89; *p* = 0.075) [37]. In the study by De Stefano et al. expression of cytoplasmic ERβ predicted poor clinical outcome in serous ovarian cancer. However, the study was smaller with 58 included ovarian cancer cases and only serous cases were included [17].

CA125 is overexpressed in the majority of serous ovarian cancers, the most common histological subtype [18]. We found a high correlation between the expression of ERα and the expression of CA125, which is in line with the findings of Sylvia et al. [38]. CA125 interacts with cell-adhesion regulators mesothelin, galectin-1 and E-cadherin and β-catenin [18, 19] and is involved in promotion of proliferation and migration [20]. Park et al. found an ERα-dependent, estrogen-induced suppression of expression and promoter activity of E-cadherin in ovarian cancer cells, whereas epithelial-mesenchymal transition-associated transcription factors, Snail and Slug, were significantly up-regulated [39]. Thus, this possible interaction between CA125 and ERα in regulation of cell adhesion should be elucidated in future studies. Also CEA has a regulative function in cell adhesion and thus an influence on metastasis of cancer cells has been suggested [21]. Another antigen selectively expressed in ovarian cancer is CA72–4. As it is highly detectable in all ovarian cancer subtypes it is another effective tumor marker in ovarian cancer [22]. However, its function in carcinogenesis of ovarian cancer is still unknown. We were able to show a high correlation between the expression of nuclear ERβ and CEA as well as CA72–4. Moreover, we observed a significant correlation between expression of ERα and PR **(**Additional file 2), which is in line with studies published earlier [28, 40].

As discussed above, the role of ERβ as a tumor suppressor in ovarian cancers is still controversial. Our data and those published by others clearly suggest a contradictory role of nuclear and cytoplasmic ERβ. Our results demonstrating an improvement of overall survival for patients with ovarian cancers expressing cytoplasmic ERβ corroborate a tumor suppressive role of ERβ. In contrast, nuclear expression of this receptor previously has been reported to shorten progression-free survival [37]. This is in accordance with our finding that expression of nuclear ERβ is associated with tumor markers CEA and CA72–4. Different binding partners depending on the subcellular location of ERβ might explain these effects [23]. Our data suggest that future studies should further assess the different roles of nuclear and cytoplasmic ERβ in ovarian cancer as previous controversial results might be due to a distinct function of ERβ depending on its location.

## Conclusions

To our knowledge, we are the first to show a correlation between nuclear ERβ and CEA as well as CA72–4 expression in ovarian cancer. Moreover, our data support the role of ERβ as a tumor suppressor in ovarian cancer, as our survival analyses show a significant benefit in overall survival of patients with tumors expressing cytoplasmic ERβ. Further studies will be necessary to examine the relevance of our data in the clinical setting.

## Additional files

Additional file 1: Antibodies used in this study. (DOCX 12 kb)

Additional file 2: Correlation of ERα expression in ovarian cancer with expression of cancer-associated genes and PR. (DOCX 19 kb)

## Abbreviations

CA125: Cancer antigen 125
CEA: Carcinoembryonic antigen
DAB: 3,3′-diaminobenzidine
ER: Estrogen receptor
IRS: Immunoreactivity score
PR: Progesterone receptor
TMA: Tissue microarray
FIGO: International Federation of Gynaecologists and Obstetricians
