# Supplementary material for: Estrogen receptor β is associated with expression of cancer associated genes and survival in ovarian cancer
Source: BMC Cancer. 2018 Oct 16;18:981. doi: 10.1186/s12885-018-4898-0 (PMC6192185; doi:10.1186/s12885-018-4898-0)
Supplement: Supplementary file 1 — Antibodies used in this study. (DOCX 12 kb) [file 12885_2018_4898_MOESM1_ESM.docx]

**Additional file 1:** **Antibodies used in this study**

| **Marker/Protein** | **Antibody Clone** | **Pretreatment** | **Dilution** | **Pattern** |
| --- | --- | --- | --- | --- |
| ERβ | PPG5/10 (Novus Biologicals) | None | 1:20 | nuclear/cytoplasmic |
| ERα | 6F11 (Novocastra) | CC1 64' | 1:35 | nuclear |
| CA-125 | OC125 (Cell Marque) | CC1 52' | 1:1 | cytoplasmic/membranous |
| CEA | A 0115 (Dako) | P1 8' | 1:500 | cytoplasmic |
| CA72.4 | B72.3 (Alexis Biochemicals) | CC1 36' | 1:50 | cytoplasmic |
| EGFR | E30 (Dako) | P1 4' | 1:100 | membranous |
| p53 | sc-263 (Santa Cruz) | CC1 36' | 1:2000 | nuclear |
| Ki-67 | MIB-1/M7240 (Dako) | CC1 64' | 1:100 | nuclear |
| PR | NCL-L-PGR-312 (Clone 16) (Novocastra) | CC1 64' | 1:50 | nuclear |
| Her2/neu | A0485 (Dako) | CC1 36' | 1:250 | membranous |

CC1: tris-EDTA borate buffer pH 8.0-8.5 at 95°C
P1: protease 1 (highest level) at 36°C
