# Supplementary material for: Estrogen receptor β is associated with expression of cancer associated genes and survival in ovarian cancer
Source: BMC Cancer. 2018 Oct 16;18:981. doi: 10.1186/s12885-018-4898-0 (PMC6192185; doi:10.1186/s12885-018-4898-0)
Supplement: Supplementary file 2 — Correlation of ERα expression in ovarian cancer with expression of cancer-associated genes and PR. (DOCX 19 kb) [file 12885_2018_4898_MOESM2_ESM.docx]

**Additional file 2: Correlation of ERα expression in ovarian cancer with expression of cancer-associated genes and PR**

Correlation of ERα expression with expression of ovarian cancer markers CA125, CEA, CA72-4 and with EGFR, HER2, Ki-67, P53 and PR were calculated for all ovarian cancers (“all”) and serous ovarian cancers (“serous”) using the Spearman correlation coefficient. P-values below 0.05 were considered statistically significant. (CI: confidence interval). Statistically significant associations were indicated in bold font.

|  |  | **CA125** | **CEA** | **CA72-4** | **EGFR** | **HER2** | **Ki-67** | **P53** | **PR** |
| --- | --- | --- | --- | --- | --- | --- | --- | --- | --- |
| **All** | Spearman r | **0.269** | 0.028 | 0.023 | -0.011 | 0.042 | -0.128 | -0.02 | **0.36** |
|  | 95% CI | **0.119**  **- 0.407** | -0.129  - 0.184 | -0.134  - 0.178 | -0.169  - 0.148 | -0.114  - 0.197 | -0.284  - 0.0337 | -0.176  - 0.137 | **0.214**  **- 0.491** |
|  | p value | **0.0004** | n.s. | n.s. | n.s. | n.s. | n.s. | n.s. | **< 0.0001** |
| **Serous** | Spearman r | **0.209** | 0.075 | 0.079 | 0.062 | 0.052 | -0.117 | 0.01 | **0.292** |
|  | 95% CI | **0.0346**  **- 0.37** | -0.104  - 0.249 | -0.099  - 0.252 | -0.119  - 0.238 | -0.126  - 0.226 | -0.293  - 0.067 | -0.168  - 0.187 | **0.119**  **- 0.447** |
|  | p value | **0.0160** | n.s. | n.s. | n.s. | n.s. | n.s. | n.s. | **0.0009** |
